# Classic ketogenic diet in parenteral nutrition in a GLUT1DS patient: Doing more with less in an acute surgical setting

**DOI:** 10.3389/fnut.2023.1114386

**Published:** 2023-02-15

**Authors:** Valentina De Giorgis, Cinzia Ferraris, Mario Leo Brena, Giorgio Farris, Valerio Gentilino, Monica Guglielmetti, Claudia Marazzi, Ludovica Pasca, Claudia Trentani, Anna Tagliabue, Costanza Varesio

**Affiliations:** ^1^Department of Child Neurology and Psychiatry, IRCCS Mondino Foundation, Member of ERN-EpiCARE, Pavia, Italy; ^2^Department of Brain and Behavioral Sciences, University of Pavia, Pavia, Italy; ^3^Human Nutrition and Eating Disorder Research Center, Department of Public Health, Experimental and Forensic Medicine University of Pavia, Pavia, Italy; ^4^Unit of Pediatric Surgery, Woman and Child Department, Filippo Del Ponte Hospital - ASST Sette Laghi, Varese, Italy

**Keywords:** GLUT1 deficiency syndrome, classic ketogenic diet, ketone bodies, parenteral nutrition, appendicitis, surgery

## Abstract

Ketogenic Dietary Treatments (KDTs) are to date the gold-standard treatment for glucose transporter type 1 (GLUT1) deficiency syndrome. Administration of KDTs is generally *per os*; however, in some conditions including the acute gastro-enteric post-surgical setting, short-term parenteral (PN) administration might be needed. We report the case of a 14-year-old GLUT1DS patient, following classic KDT for many years, who underwent urgent laparoscopic appendectomy. PN-KDT was required, after 1 day of fasting. No *ad hoc* PN-KDTs products were available and the patient received infusions of OLIMEL N4 (Baxter). On the sixth day postoperatively enteral nutrition was progressively reintroduced. The outcome was optimal with rapid recovery and no exacerbation of neurological manifestations. Our patient is the first pediatric patient with GLUT1DS in chronic treatment with KDT efficiently treated with exclusive PN for five days. This case reports on real-word management and the ideal recommendations for PN-KDT in an acute surgical setting.

## 1. Introduction

Glucose Transporter Type 1 Syndrome (GLUT1DS) is a rare genetically determined neurometabolic disorder causing impaired glucose transport through the blood-brain barrier. The GLUT1DS key clinical features are mainly represented by eye-head movement abnormalities, epileptic seizures, neurodevelopmental impairment, deceleration of head growth, and movement disorders (1). Ketogenic dietary therapies (KDTs) -high-fat, carbohydrate-restricted, adequate proteins- are, to date, the gold standard treatment for the syndrome (2): ketone bodies can cross the blood-brain barrier and can be used as an alternative fuel for brain metabolism.

Ketogenic dietary therapies are generally administered *per os*; however, a variety of acute conditions, causing transient intestinal failure or in an immediate gastro-enteric post-surgical setting, might require complete bowel rest, thus requiring short-term parenteral administration as a bridge to the reintroduction of enteral KDTs (3). However, there are currently extremely limited data, with less than 40 patients described up-to-date in literature, focusing on the feasibility and efficacy of parenteral nutrition (PN) KDTs (PN-KDTs) (4).

Herein, we present the first pediatric patient with GLUT1DS in chronic treatment with classic KDT (cKDT) efficiently treated with exclusive PN for 5 days.

## 2. Case report

We report the case of a 14 years old boy affected by GLUT1DS, whose clinical picture encompassed mild cognitive impairment, generalized epileptic seizures (atypical absences) at the age of three, partially controlled with valproic acid, and a paroxysmal movement disorder (characterized by paroxysmal exercised induced dystonia and episodes with choreoathetosis features). At the age of 5 years, genetic diagnosis of GLUT1DS was provided, and a classic ketogenic diet (cKD) with a 2.6:1 (fat to protein + carbohydrate) ratio was administered with complete resolution of epileptic seizures and movement disorder for more than 9 years. At the time of the event, the patient was not receiving any concomitant treatment. During the years, regular ketone monitoring revealed mean values of beta-hydroxybutyrate ranging from 1.6 to 2 mmol/L. Adequate minerals and vitamin supplementation were associated with KDT. The patient was regularly followed at Mondino Neurological Institute (Pavia) to monitor neurological and nutritional aspects, according to recent guidelines for KDTs (2).

No adverse events were derived from KDT implementation. At the last follow-up 2 months before the acute event, a stable clinical picture was documented, with an EEG substantially within normal limits.

Due to abdominal pain and vomiting, the patient was admitted to the Pediatric Emergency Department of Del Ponte Hospital (Varese). After surgical evaluation, the patient underwent laparoscopic appendectomy, which revealed a clinical picture of acute perforated gangrenous appendicitis with diffuse peritonitis. Broad-spectrum antibiotic combination therapy saline solution was administered as per internal Hospital protocol. The patient recovered well from surgery; however, after 1 day of fasting, based on daily consultation between surgeons and the patient’s referral Keto-Team, PN-KDT was started as the boy was unable to tolerate enteral nutrition postoperatively. Data on parenteral and enteral nutrition management are shown in Table 1. PN was provided through a central venous catheter. Given the unavailability of ketogenic formulated products and of *ad hoc* galenic formulation production for PN-KDT in an emergency setting, where treatments with KDTs are not commonly used, the patient received infusion of OLIMEL N4 (Baxter). From the second to the fifth day of PN only OLIMEL N4 solution was administered, and the quantity was increased from 500 ml/day (equal to 560 kcal/day) to 1,440 ml/day (equal to 1,008 kcal). Since OLIMEL N4 (Baxter) is a non-ketogenic product, a rapid and conspicuous drop in ketonemia was expected. To partially overcome this inconvenience, the caloric intake was calculated to be lower than expected, to stimulate fasting ketones production. Hydration was administered according to water balance. Ketonemia was monitored every 6 h, with ketone levels in range (2.7 mmol/L) during the first day of fasting, and subsequent reduction ranging from 1 to 1.2 mmol/L. On the sixth day, enteral nutrition was progressively reintroduced through a ketogenic formulated product (Ketocal powder 3:1, Nutricia) (Table 1) *via* a nasogastric (NG) tube. The product was given in doses of 30 g, diluted in 120 ml of water, three times a day in addition to the PN-KDT. In the following 2 days, PN-KDT was progressively reduced, and at the same time, the NG-tube feeding formula was increased to 45 g three and four times a day, respectively (Table 1). Solid food (cKD with a 2.6:1 ratio) was reintroduced after 9 days from surgery and was well tolerated. Close monitoring of serum electrolytes and metabolic profile was conducted without detecting clinically noteworthy changes.

**TABLE 1:** Timeline of Practical plan of Parenteral and Enteral Nutrition (PEN) management.

	Type of feeding			Macronutrients				Total calories	Ketogenic ratio
		**Velocity**	**Quantity**	**Total lipids (g)**	**Total AA (g)**	**Nitrogen (g)**	**Total glucose (g)**		
1st day	Fasting								
2nd day	PN-Olimel N4	30 ml/h	800 ml/day	24	2,026	3.2	60	560	0.30
3rd day	PN-Olimel N4	40 ml/h	960 ml/day	288	2,432	3.84	72	672	0.30
4th day	PN-Olimel N4	50 ml/h	1,200 ml/day	36	304	4.8	90	840	0.30
5th day	PN-Olimel N4	60 ml/h	1,440 ml/day	432	3,648	5.76	108	1,008	0.30
6th day	PN-Olimel N4	80 ml/h	1,500 ml/day	45	38	6	11,25	16,899	1.67
	NG-tube	3 times/day	30 g	10,674	5,186		11,898		
7th day	PN-Olimel N4	60 ml/h	1,440 ml/day			5.76		19,6785	1.67
	NG-tube	3 times/day	45 g	13,581	5,727		11,772		
8th day	PN-Olimel N4	40 ml/h	960 ml/day			3.84		19,518	1.67
	NG-tube	4 times/day	45 g	15,228	5,204		8,496		
9th day	Solid food *per os*			2,088	456		341	2,230	2.62

No exacerbation of paroxysmal neurological symptoms was observed during PN, nor at the time of enteral feeding reintroduction. At subsequent follow-up 2 months and 6 months after surgery, the patient depicted good general health, no recurrence of paroxysmal neurological events, substantially normal EEG, normal blood tests, and blood glucose and ketonemia levels comparable to those before surgery.

## 3. Discussion

Up to now, PN-KDTs described in the literature were only administered in hospitals specialized in the management of KDTs, when the diet needed to be initiated and an enteral approach was not feasible due to an acute condition as, for example, refractory seizures or in patients with drug-resistant epilepsies, already on KDT, in whom an acute condition impaired enteral feeding (3). Some attempts to systematize the application and management of PN-KDTs have been proposed in recent years by van der Louw et al. (4) and by Dressler et al. (5). However, in Italy, only a few Centers are equipped for the chronic and acute management of KDTs. In a recent Italian web-based survey from our group aiming at investigating caregivers’ perceptions about the management of a patient undergoing the cKD in the acute settings, it was highlighted that in a surgical context, the fasting duration, support therapies, and feeding after gastrointestinal surgical procedures should be considered as unsolved themes to be addressed by experts (6).

To the best of our actual knowledge, we reported the first GLUT1Ds patient receiving short-term parenteral nutrition in the immediate post-surgical period, in a setting where PN-KDTs products were unavailable. What we have done was quite different from the recommendations, reported in Table 2, due to the urgency of the condition and the unfavorable circumstances, but it was associated to prompt recovery of ketonemia at the typical pre-surgery values.

**TABLE 2:** Timeline of Theoretical plan for Parenteral and Enteral Nutrition (PEN) management.

	PN-Products to be used			Macronutrients				Total calories	Ketogenic ratio
		**Velocity**	**Quantity**	**Lipid (g)**	**Amino Acid (g)**	**Nitrogen**	**Glucose (g)**		
1st day	Aminoplasmal 10%	10 ml/h	300 ml		30			160	
	Glucose 5%	12 ml/h	200 ml				10		
	Fluids		as required						
2nd day	Aminoplasmal 10%	10 ml/h	300 ml		30			1,150	2.00
	Glucose 5%	25 ml/h	400 ml				20		
	Lipofundin 20%	32 ml/h	500 ml	100					
	Fluids		As required						
3rd day	Aminoplasmal 10%	22 ml/h	350 ml		35			1,427	2.08
	Glucose 5%	31 ml/h	500 ml				25		
	Lipofundin 20%	39 ml/h	625 ml	125					
	Fluids		As required						
4th day	Aminoplasmal 10%	26 ml/h	400 ml		40			1,685	2.31
	Glucose 5%	31 ml/h	500 ml				25		
	Lipofundin 20%	47 ml/h	750 ml	150					
	Fluids		As required						
5th day	Aminoplasmal 10%	28 ml/h	450 ml		45			1,943	2.50
	Glucose 5%	31 ml/h	500 ml				25		
	Lipofundin 20%	54 ml/h	875 ml	175					

It is generally accepted that the introduction of parenteral nutrition should be considered when the pediatric patient cannot receive enteral feeding for more than 48 h (7). In such a context, particularly when the patient is already receiving KDTs as the only treatment, as in many GLUT1DS patients, early contact with the patient’s referring keto-team is essential to establish an effective shared and coordinated management. If the patient had been admitted to a Reference Center for the ketogenic diet, individualized feeding and hydration plans would have been conceived considering his caloric and fluid needs. In this scenario, a theoretical plan for managing our patient is presented in Table 2. However, theoretical assumptions often clash with contingent reality in critical conditions, as underlined by our case. In particular, although theoretically all fluids containing dextrose should ideally be avoided, from a practical standpoint, management of our case supports the hypothesis that in acute settings, when preparations for PN-KDTs are not available, standard intravenous solutions commercially available and promptly disposable at local hospitals might be at least an acceptable compromise.

As suggested by our case, their application should be limited to the very short period of the acute condition, and a change into enteral nutrition, with a slow increase in the amount of enteral feeding, should be promoted as soon as possible.

Notably, short-term parenteral nutrition with commercially available formulations was revealed to be safe and well-tolerated in our patient. In addition, control of paroxysmal symptoms (both epileptic seizures and paroxysmal moment disorder) was maintained during the transition from enteral to parenteral nutrition and when switching back to enteral nutrition.

Our patient remained free from paroxysmal symptoms despite reducing ketosis from baseline. Such a condition was observed even in the case series reported by Armeno et al. (3), thus raising the question about the need to maintain a high ketogenic ratio during PN-KDT. Although suboptimal, we believe that the ketonemia values presented by our patient during PN may have contributed to the non-occurrence of side effects. The most commonly reported side effects of PN-KDTs include increased lipids profile, insufficient ketosis, or hypoglycemia, whereas less frequently altered liver, and pancreatic functions have been described; they are usually transient and reversible by switching to enteral feeding (8). Therefore, careful monitoring of glucose and ketones levels, along with electrolytes, lipid profile, and liver and pancreatic enzymes, is mandatory (4). As stated by Van der Louw et al. (4), adverse events can be avoided by limiting parenteral nutrition to a very short period of the acute condition and promoting a change into enteral nutrition, with a slow increase in the amount of enteral feeding as soon as possible. Our case seem to confirm this observation.

In conclusion, our experience supports the idea that PN may be considered an efficient temporary solution toward enteral KD in those GLUT1DS patients who are temporarily prevented from enteral feeding due to acute medical or surgical gastroenteric pathologies. However, for proper management and monitoring of dietary aspects in such tricky conditions, the close and continuous collaboration of a multidisciplinary team daily supporting and sharing expertise with colleagues operating in the frontline emergency setting is essential.

## Data availability statement

The original contributions presented in this study are included in the article/supplementary material, further inquiries can be directed to the corresponding author/s.

## Ethics statement

The studies involving human participants were reviewed and approved by Pavia Ethical Committee. Written informed consent to participate in this study was provided by the participants’ legal guardian/next of kin.

## Author contributions

CV, CF, AT, and VD contributed to the conception and design of the report. VG, MB, GF, MG, CM, LP, and CT contributed to the acquisition, analysis, and interpretation of the data. All authors drafted the manuscript, critically revised the manuscript, agreed to be fully accountable for ensuring the integrity and accuracy of the work, and read and approved the final manuscript.

## References

[1] Klepper J Akman C Armeno M Auvin S Cervenka M Cross H Glut1 deficiency syndrome (Glut1DS): state of the art in 2020 and recommendations of the international Glut1DS study group. *Epilepsia Open.* (2020) 5:354–65. 32913944 10.1002/epi4.12414 PMC7469861

[2] Kossoff E Zupec-Kania B Auvin S Ballaban-Gil K Christina Bergqvist A Blackford R Optimal clinical management of children receiving dietary therapies for epilepsy: updated recommendations of the international ketogenic diet study group. *Epilepsia Open.* (2018) 3:175–92. 10.1002/epi4.12225 29881797 PMC5983110

[3] Armeno M Verini A Araujo M Reyes G Caraballo R. Ketogenic parenteral nutrition in three paediatric patients with epilepsy with migrating focal seizures. *Epileptic Disord.* (2019) 21:443–8. 10.1684/epd.2019.1095 31617493

[4] Van der Louw E Aldaz V Harvey J Roan M van den Hurk D Cross J Optimal clinical management of children receiving ketogenic parenteral nutrition: a clinical practice guide. *Dev Med Child Neurol.* (2020) 62:48–56. 10.1111/dmcn.14306 31313290 PMC6916385

[5] Dressler A Haiden N Trimmel-Schwahofer P Benninger F Samueli S Gröppel G Ketogenic parenteral nutrition in 17 pediatric patients with epilepsy. *Epilepsia Open.* (2017) 3:30–9. 10.1002/epi4.1208 29588985 PMC5839306

[6] Pasca L Varesio C Ferraris C Guglielmetti M Trentani C Tagliabue A Families’ perception of classic ketogenic diet management in acute medical conditions: a web-based survey. *Nutrients.* (2020) 12:2920. 10.3390/nu12102920 32987704 PMC7598657

[7] Skillman H Wischmeyer P. Nutrition therapy in critically ill infants and children. *JPEN J Parenter Enteral Nutr.* (2008) 32:520–34. 10.1177/0148607108322398 18753390

[8] Lin K Lin J Wang H. Application of ketogenic diets for pediatric neurocritical care. *Biomed J.* (2020) 43:218–25. 10.1016/j.bj.2020.02.002 32641260 PMC7424092

